# Investigating the links between questionable research practices, scientific norms and organisational culture

**DOI:** 10.1186/s41073-024-00151-x

**Published:** 2024-10-14

**Authors:** Robin Brooker, Nick Allum

**Affiliations:** University of Essex, Wivenhoe Park, Colchester, CO4 3SQ UK

**Keywords:** Research Integrity, Questionable Research Practices, Scientific Norms

## Abstract

**Background:**

This study investigates the determinants of engagement in questionable research practices (QRPs), focusing on both individual-level factors (such as scholarly field, commitment to scientific norms, gender, contract type, and career stage) and institution-level factors (including industry type, researchers' perceptions of their research culture, and awareness of institutional policies on research integrity).

**Methods:**

Using a multi-level modelling approach, we analyse data from an international survey of researchers working across disciplinary fields to estimate the effect of these factors on QRP engagement.

**Results:**

Our findings indicate that contract type, career stage, academic field, adherence to scientific norms and gender significantly predict QRP engagement. At the institution level, factors such as being outside of a collegial culture and experiencing harmful publication pressure, and the presence of safeguards against integrity breaches have small associations. Only a minimal amount of variance in QRP engagement is attributable to differences between institutions and countries.

**Conclusions:**

We discuss the implications of these findings for developing effective interventions to reduce QRPs, highlighting the importance of addressing both individual and institutional factors in efforts to foster research integrity.

**Supplementary Information:**

The online version contains supplementary material available at 10.1186/s41073-024-00151-x.

## Introduction

Questionable Research Practices (QRPs) refer to suboptimal research practices that exist in an area of ethical ambiguity between sound scientific conduct and outright scientific misconduct (i.e., fabrication, falsification, and plagiarism) [1–5]. Given their inherent ethical ambiguity, these practices may be seen as warranted or defensible in certain contexts and some of the time [1]. These practices include hypothesising after results are known (termed 'Harking') [6], including authors on publications who have not contributed sufficiently to warrant authorship and selectively reporting study outcomes ('cherry picking'). While these practices are perhaps insignificant in isolation, their cumulative impact over time can adversely affect science by undermining the reliability and validity of scientific knowledge. For example, the various methodological decisions that are inherent to the research process, known as 'researcher degrees of freedom,' have been found to elevate Type I error rates [2; p. 1359]. QRPs lead to a skewed scientific literature that inevitably undermines efficiency and impedes the scientific process. That is, prolonging support for empirically untenable theories that should be revised or discarded. Downstream, these practices at the very least contribute to wasted resources and at most lead to the canonisation of erroneous scientific claims [7], the compromising of science-informed policy and interventions, and an erosion of public trust in science [7].

QRPs are of particular concern given the frequency at which researchers engage in them. Martinson et al. [8] found that 33% of respondents in their survey admitted to having engaged in at least one QRP or research misconduct within the last three years, while John and colleagues [1], using an anonymous elicitation survey format combined with incentives for truth telling, identified prevalence estimates up to 100% for some QRPs amongst psychologists. Like Martinson and colleagues [8], an early meta-analytic study by Fanelli [9] found that approximately 34% of respondents admitted to engaging in at least one QRP at some point during their academic career. However, a recent meta-analytic study by Xie and colleagues [10] found a more modest estimate, with prevalence rates of around 12.5%. While there is evidence to suggest that the interpretation of survey data can sometimes lead to exaggerated prevalence estimates, as in the case with John and colleagues' [1] study (see, Fiedler & Schwarz) [11], the existence of prevalence rates that are far from non-zero is a matter of considerable concern.

Given the far-reaching downstream consequences and frequency of occurrence, a growing number of research publications, which have informed reform initiatives and interventional and educational strategies, have sought to elucidate and capture the factors that motivate engagement in suboptimal research practices. Proposed determinants of QRPs can be categorised across three distinct, but interrelated, strata: Individual-level factors that are intrinsic to researchers, organisational-level factors pertaining to research institutions, and systemic-level factors inherent within the broader research ecosystem. The relationship between QRPs and determinants at all levels remains largely unclear, with scant empirical literature investigating the antecedents of QRPs.

### Individual factors

At the individual-level, cognitive biases when navigating researcher degrees of freedom [1, 12, 13] and competency shortfalls in research ethics [14, 15], research methodology [16, 17] and data analysis [18] have been postulated as potential explanatory factors for engaging in QRPs. The personality traits of agreeableness and conscientiousness have been found to be inversely associated with self-reported prevalence of QRPs in survey research [19, 20], while the trait of 'Machiavellianism' has been identified as a potential risk factor for research misbehaviour [21]. Janke and colleagues [22] found that among a sample of psychologists stronger appearance approach goals (i.e., striving for skill demonstration) and lower learning approach goals (i.e., striving for skill development) predicted greater engagement in self-reported QRPs. Moreover, Sacco and colleagues [23] found that researchers judged QRPs to be ethically defensible and were more willing to engage in them where the practice was perceived to be professionally appropriate.

A researcher's commitment to normative ideals of science has the potential to subvert other motivating factors. Robert Merton [24] argued that scientists adhere to a set of informal cultural norms, including universalism (evaluating claims based on pre-established impersonal criteria, rather than nationality, ethnicity, gender or professional affiliation), communalism (scientific findings belong to the entire scientific community), disinterestedness (scientists' work should not be influenced by personal or monetary bias, and they should work for the benefit of science), and organised scepticism (assessment of research should be based on impersonal critical scrutiny). Although recent investigations into the extent to which scientists subscribe to these norms are scant, where surveys have been carried out, primarily in specific disciplines, evidence has shown that adherence, although not universal, is widespread [25–28]. Feasibly, a commitment to these norms may mitigate the influence of competing influences, such as organisational and systemic pressures to cut corners to remain competitive. However, empirical inquiry investigating how commitment to the ideals of science is associated with behaviour is noticeably absent, except for a recent survey of researchers in The Netherlands that found that commitment to the normative ideals of science was one of the strongest predictors for not engaging in QRPs [29].

The relationship between QRP engagement and other individual-level factors, such as gender, career stage and years since obtaining a PhD are even less clear. Preliminary evidence from a sample of researchers in The Netherlands found that women are less likely to engage in QRPs [29], while a more recent study by Schneider et al. [19] found that male Danish respondents reported slightly higher QRP prevalence rates compared to female respondents. This effect was less clear among a non-Danish sample. In the same study, Schneider and colleagues found that academic experience (years since obtaining a PhD) was associated with less QRP engagement. Relatedly, Gopalakrishna et al. [29] found that PhD candidates and junior researchers are more likely to engage in at least one QRP frequently compared to postdocs and assistant professors. However, no statistically significant relationship between academic rank and average engagement in QRPs was found. Research showing disciplinary field differences in QRP engagement additionally present conflicting findings (e.g. [10, 19]).

### Organisational and systemic factors

At the system level, it is widely believed that the misalignment between incentive structures and the principles of scientific integrity play a significant role in influencing the engagement in QRPs (e.g., [8, 30, 31]). The favouring of the aesthetic quality of results (e.g., novelty and statistical significance) by journal editors over the reproducibility and robustness of scientific findings arguably leads to a culture that prioritises eye-catching outcomes at the expense of methodological soundness. This is demonstrated by the conspicuous absence of null findings in the scientific literature. Chavalarias and colleagues [32] found that statistically significant results (i.e., *p*-value < 0.05) account for approximately 96% of published abstracts in MEDLINE and full-text articles in PubMed Central. On this basis, there have been calls for journals to increase publication of negative results [33, 34] and confirmatory findings [35] as well as improved systems and platforms to facilitate reproducibility [36]. Moreover, registered reports, which involve journal editors agreeing to publish unknown results based on a proposed design and methodological rigour, are increasingly being utilised (e.g., in Cortex and Comprehensive Results in Social Psychology) to address the bias towards statistically significant findings.

At the institutional-level, simple metrics, such as frequency of publication and journal impact factors, are used when assessing for hiring, promotion, tenure, and permanency. These institutional metrics combined with the systemic bias that favours the aesthetic quality of research, creates a competitive and high-pressure culture, where researchers are incentivised to cut corners to remain competitive. Several studies have found self-reported publication pressure to be positively associated with self-reported use of QRPs [19, 29, 37]. Research in the Netherlands has shown that this pressure is felt by researchers operating across fields, with approximately 54% of surveyed professors feeling that the pressure to publish is excessive [38]. On this basis, there have been calls for broad institutional revisions in how researchers are hired, evaluated, and promoted [39, 40], with a reduced emphasis on journal impact factors and other commonly used metrics [41]. Similarly, Principles 4 and 5 of the Hong Kong Manifesto endorsed by the 6th World Conference on Research Integrity call for reforms in the way researchers are assessed and valued, focusing on valuing a broader range of researcher contributions, including replication attempts, peer review, mentoring and knowledge transfer [42]. Empirically, local culture has been found to be associated with QRP prevalence [19], whereby research cultures that promote quality and rigorous research, and reward integrity, appear to counter systemic use of QRPs to some degree.

These organisational and system level factors may interact with individual-level motivations, such as the personal-economic and career-specific motivations of researchers. For example, institutions that pressure scientists to increase their research output (e.g., using publication metrics) are most likely especially influential when scientists are motivated by job permanency, promotions, pay rises and social approbation. That is, the susceptibility to negative impacts (e.g., systemic influences) on behaviours of researchers may not be uniformly distributed across individuals [19].

## The current study

In this study, we seek to explore the relative influence of selected individual-level, organisational and systemic level factors on engagement in QRPs, using data from an international survey of researchers operating across scholarly fields. The factors suggested as explanations for engagement in QRPs in the extant research literature are grounded in limited and disjointed evidence. We aim to bring a more coherent and broader perspective by exploiting a comprehensive source of data not previously available. Additionally, the relative importance of individual versus organisational factors in explaining QRPs is an important, but under-explored, area of inquiry. The relative influence attributable to individual-level and organisation-level factors is important for the development of efficacious interventional strategies that appropriately target the primary motivators of QRP engagement, thus improving the efficiency, quality, and credibility of scientific research. On the one hand, if a significant proportion of variance in researcher QRP engagement is attributable to organisational-level differences, there is an impetus for further examination of institutionally specific factors and institutionally tailored solutions and policy revisions within these settings. On the other, if systematic variation in QRPs across institutions is minimal, this would suggest that individual-level factors (e.g., nurturing ideals of sound science and competencies), as well as systemic factors (e.g., eliminating systemic pressures and hyper-competition), should be the primary targets of intervention.

The International Survey on Research Integrity (IRIS), a large-N cross-sectional survey, administered to researchers in the US and Europe, presents an ideal opportunity to assess the impact of some widely postulated individual and organisational level factors in motivating QRP engagement. IRIS includes individual-level items allowing us to explore the impact of employment contract type, career stage, disciplinary field, sex, and commitment to the ideals of science on QRP engagement. It additionally includes items that measure organisational level features, including workplace type, existence of integrity training and procedures for tackling integrity breaches, whether institutions have a written statement on research integrity and perceptions of collegiality and pressure in working environment. Thus, the survey data permits us to explore the relative impact of organisational-level characteristics on QRP engagement, at least as perceived by the respondent. The ability to disaggregate research institutions additionally allows us to explore the extent to which variance in QRP engagement can be attributed to organisational and individual level differences. Moreover, while existing research typically examines a narrow set of disciplinary fields, the IRIS includes respondents representing researchers from across diverse fields.

The cross-national nature of the survey allows us to go beyond individual and organisation and examine country level differences in QRP engagement. Xie, Wang and Kong [10] found that research misconduct and QRPs appear to be higher in low- and middle-income countries (LMICs, e.g., Nigeria and India), relative to high-income countries (HICs, e.g., the UK and USA), resulting from lack of provision of standards of responsible research conduct and lack of organisation. However, there are also distinct standards, policies and cultures that could differentiate amongst HICs. For example, Allum et al., using the IRIS data, found differences between Europe and the US on a range of research integrity indicators, including QRPs. We go beyond their analysis to consider how important is the country context after accounting for individual characteristics—which may be distributed quite differently across HICs. Diverse educational systems with differing emphases placed on ethical practices, as well as differing regulatory frameworks for detecting and deterring QRP engagement are among those unobserved factors that may lead to substantial country level variance. Our analysis permits us to explore these country differences, predominantly within Europe, alongside the other explanatory variables already described.

## Data and methods

The data come from a cross-sectional international survey of researchers, administered in 2021 as part of the European Commission-funded ‘Standard Operating Procedures for Research Integrity’ (SOPs4RI) project. The online survey utilised a systematic, stratified probability sample of over 60,000 researchers from across scholarly fields. The sample comprised authors of research articles indexed in Clarivate’s Web of Science citation database. The survey protocol was pre-registered with the Center for Open Science in February 2019 (available, along with reproducibility materials at Reference [43]). Further details about IRIS are contained in Allum et al. [44] Our analytical sample is comprised of 39,699 researchers. Of these, 57.6% were male. 40.8% primarily research in the natural sciences, 14.4% in the medical sciences, 30.8% in the social sciences and 14% in the humanities. 4.5% are employed in the United States of America, 86.7% in Europe and 8.7% elsewhere.

### Variables

#### Questionable research practices

A total of eight behaviours pertaining to various aspects of the research process (e.g., publication, PhD supervision) that are widely considered to be questionable and undermining of the trustworthiness of scientific findings were generated for the purposes of the survey. Descriptions of these questionable research practices (QRPs) can be found in Table 1.

**Table 1:** Questionable research practice items from international survey on research integrity

	Questionable Research Practice (QRP) Question Wording
QRP 1	Wilfully failing to cite relevant publications that contradict your own beliefs, theories, hypotheses, methods or findings
QRP 2	When reviewing a manuscript, not investing the effort necessary to conduct a thorough review
QRP 3	Choosing not to report your findings if they could weaken or contradict your theories of hypotheses
QRP 4	Deliberately using another researcher's unpublished idea without giving credit. For example, publishing an idea voiced by a colleague at an informal meeting without giving them credit
QRP 5	In a publication, failing to disclose relevant personal, financial, political or intellectual conflicts of interests
QRP 6	Including authors on a paper who had not contributed sufficiently to the work to merit authorship
QRP 7	Inadequately supervising or mentoring junior co-workers
QRP 8	Carrying out research without getting the required ethical approval

**Fig. 1:**
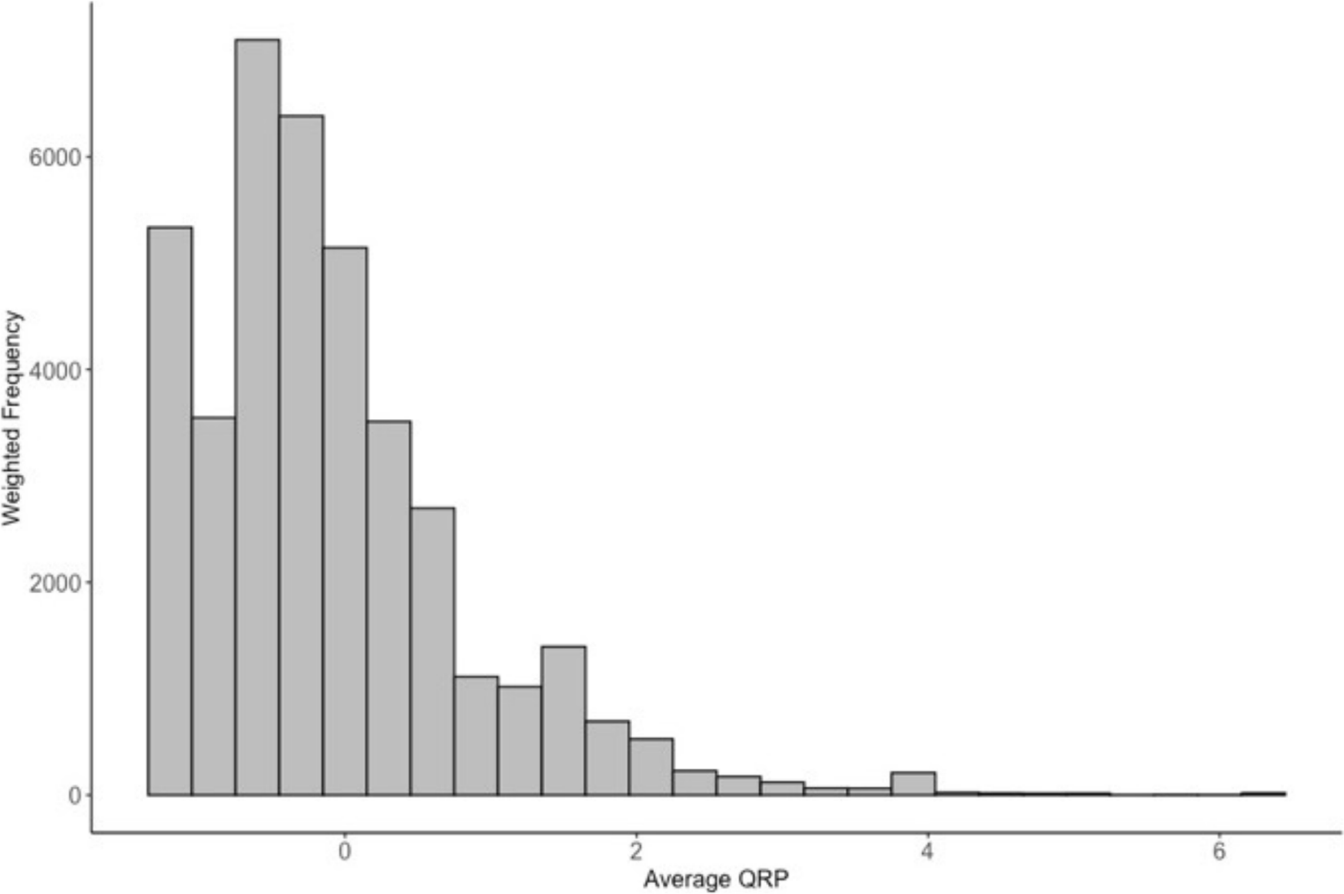
Weighted distribution of standardised mean QRP engagement for 39,699 respondents

Each QRP was accompanied with a question asking: 'thinking about your research carried out for your publications over the last three years, how often has the following occurred?', with response categories 'often', 'sometimes', 'rarely', 'never', 'does not apply'. The three-year timeframe was used to limit recall bias and to be able to state prevalence in more precise terms. The eight individual QRP item scores were aggregated for each respondent, resulting in a total score ranging from 0 to 32. This sum was then divided by the count of items that were both responded to and deemed relevant by respondent (i.e., where 'does not apply' was not selected). This scale was standardised to z-scores, ensuring each score is mean-centered and scaled by the standard deviation, with higher scores indicating greater average engagement. Operationalising 'QRP engagement' in this way is consistent with previous research that has attempted to model the relationship between various explanatory factors and QRPs (e.g., Gopalakrishna et al. [29]). Figure 1 displays the weighted distribution of our standardised mean QRP engagement (mean = -0.04, median = -0.18, standard deviation = 0.92).

#### Respondent-level explanatory factors

**Fig. 2:**
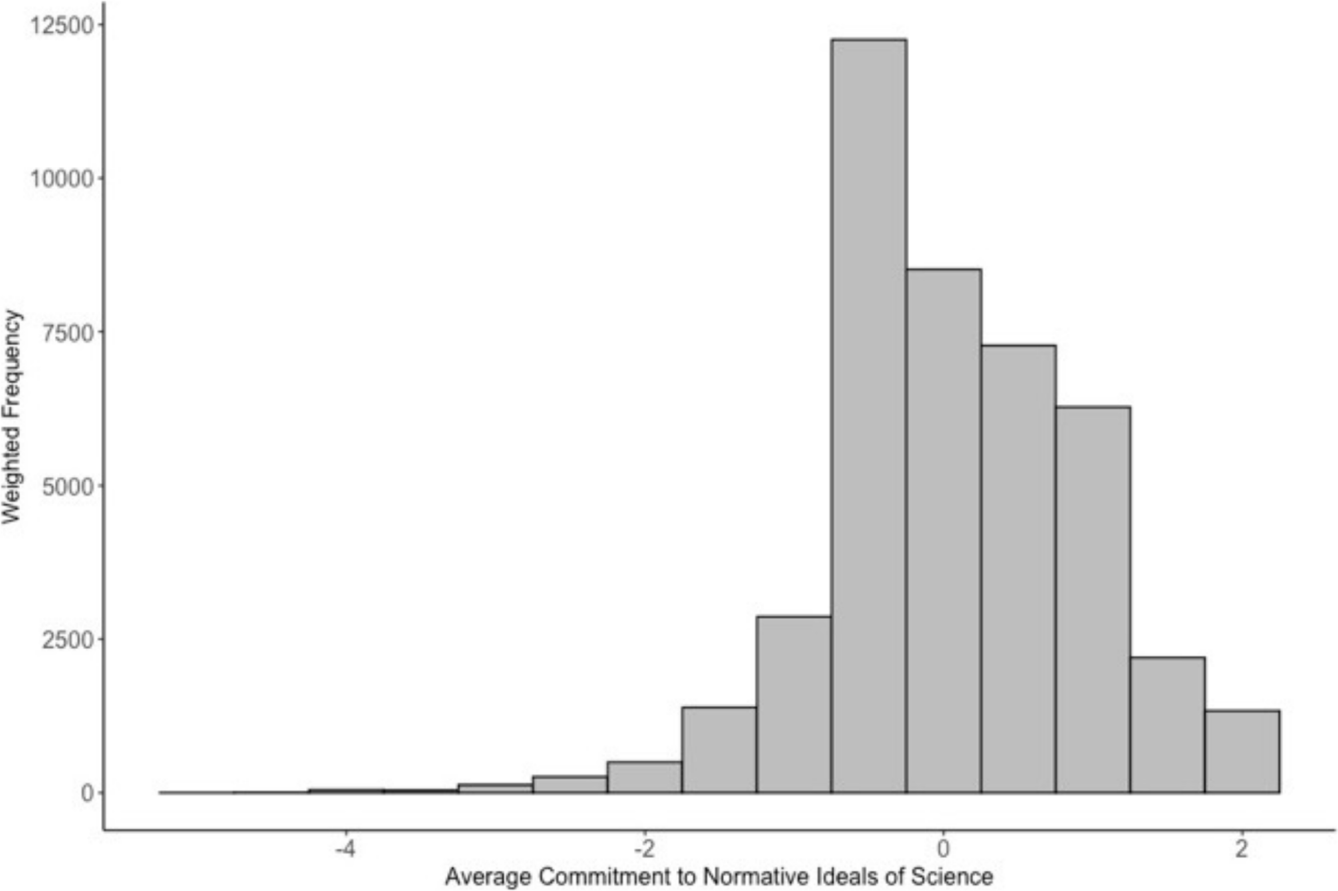
Weighted distribution of our standardised mean commitment to normative ideals of science for 39,699 respondents

A composite variable indicating scientists’ commitments to the normative ideals of science was computed based on five items assessing researchers’ values, each based on Merton's delineation of the normative ideals of science (i.e., universalism, disinterestedness, organised scepticism, and communalism). These items were adapted from Anderson [45] and Anderson et al. [25]. They described a set of behaviours, and respondents indicated whether they personally feel that these behaviours reflect how researchers should behave, with response categories anchored at 'yes, usually should' (coded 1) to 'no, never should' (coded 5). Descriptions of these normative ideals can be found in Table 2. Items 3 and 4 were reverse-coded. For each respondent, an average score was computed based on the five items. This composite variable was standardised to mean zero and standard deviation one. Higher scores indicate a greater commitment to the normative ideals of science. Figure 2 displays the weighted distribution of our standardised composite variable, which represents the average adherence to the normative ideals of science (mean = 0.06, median = 0.12, standard deviation = 0.88).

**Table 2:** Normative ideals of science items from international survey on research integrity

	Mertonian Norm	Normative Ideal of Science Question Wording
Item 1	Communalism	"Researchers should openly share new findings with colleagues"
Item 2	Organised Scepticism	"Researchers should consider all new evidence"
Item 3	Disinterestedness 1	"Intellectual work should be influenced by personal beliefs and values"
Item 4	Disinterestedness 2	"Researchers should change their research interests to access research funding"
Item 5	Universalism	"Researchers should always publish findings that are scientifically sound"

Each respondent reported their main disciplinary field and was assigned to one of four broad categories: social sciences, humanities, medical sciences (including biomedicine), and natural sciences (including technical sciences). This variable was recoded into three binary variables representing each scientific field, with 'natural sciences' omitted, as the reference category. Respondents additionally provided the type of employment contract that they are on from a pre-specified list: permanent contract, temporary contract, or no contract. We recoded this variable into two binary variables with permanent contract omitted as reference category. Respondents indicated the stage of their career, selecting from either early-career, mid-career, late-career or retired. This variable was also recoded into three dummy variables, with the reference category early-career. Each respondent reported their sex, with response categories male, female and prefer not to say. Those stating prefer not to say (*N* = 1109) were removed from the analysis. This was recoded as a dummy variable with male omitted as the reference category.

#### Organisation level explanatory factors

Each participant was asked to identify their workplace type from six sectors: academia, industry, not-for-profit research institute, government research centre, healthcare setting, or other. We recoded this variable into five indicator variables, using academia as the reference category. Respondents were also presented with a series of descriptions characterising what could be regarded as a functionally optimal working environment and were asked the extent to which the descriptions resemble their own working environment. The items selected for use in our analysis were the presence of adequate integrity training (i.e., ‘training in research integrity is provided to all researchers, at all career stages, by qualified trainers’), provision for handling integrity breaches (i.e., ‘researchers can consult a qualified person in confidence with any research integrity concerns. Breaches are detected and sanctioned in a fair and standardized way, protecting both whistleblowers and those accused of misconduct’) and a positive working culture (i.e., ‘collegial, and without harmful publication pressure, detrimental power imbalances or conflict’). These were measured on a five-point scale, anchored at ‘resembles my environment very closely’ (coded 1) and ‘resembles my environment not at all closely’ (coded 5). We reverse coded this variable so that higher scores indicated that the description is more reflective of their working environment. The variables were mean-centred and standardised to z scores. Additionally, respondents were asked to state whether their institution has a written statement on research integrity, with three answer categories: 'yes', 'no' and 'I don't know'. We recoded this variable into two dummy variables, with 'yes' as the reference category.

#### Contextual variables

Integral to the present research is the degree to which variability in QRP engagement is associated with differences between organisations and countries. We use the email addresses of survey respondents to create a variable indexing organisations, based on their domain name (e.g. @harvard.edu). Generic email addresses were identified using a non-exhaustive list of the most used general email addresses (e.g. Gmail, Hotmail). We deleted cases where the domain name was not associated with a research-producing organisation. In total, 9,304 non-institutional email addresses were removed, leaving our analytical sample containing a total of 7,666 unique organisations. Figure S1 in the supplementary material presents the frequency distribution and descriptive statistics for organisation. The second contextual variable, country, was based on the self-reported country of employment. We focused on the 'country of employment' as it reflects the researcher's location and, consequently, their organisational setting. The final analytical sample consisted of respondents employed across 34 countries. These were predominantly in Europe.

#### Interaction terms

To assess the extent to which a researcher's scientific values may act as a safeguard against workplace pressures and non-collegial working environments, thus reducing engagement in QRPs, we specify a cross-level interaction, interacting commitment to the normative ideals of science with working environment. We additionally test whether a researcher's working environment has more of an influence on research conduct for those early in their career and those on non-permanent contracts, who potentially have more to gain from engaging in suboptimal practices. On this basis, we create several interaction terms, interacting the working environment on our career stage and employment contract indicator variables. Moreover, we expect the influence of a less collegial and more competitive working environment on engagement in QRPs to be reduced where integrity training is sufficient, there are procedures (incl. firmer penalties) for handling contraventions of good research practice, and where the researcher is aware of the institutions commitment to ensuring research integrity (i.e., by way of awareness of a written statement on research integrity). Therefore, we specify several interaction terms, interacting working environment with a) existence of integrity training, b) procedures for handling integrity breaches, c) there being no written statement on research integrity and d) lack of awareness of whether there is a written statement on research integrity.

The presence of integrity training, along with suitable procedures and personnel for addressing integrity violations, could potentially support researchers in adhering to Mertonian scientific values. As a result, we define interaction terms for the commitment to these ideals of science in relation to a) integrity training, and b) handling of integrity breaches. Finally, the relationship between having an awareness of a written statement on research integrity and QRP engagement will plausibly be stronger for those who are more strongly committed to the normative ideals of science. Conversely, we expect the presence of a research integrity statement to matter less, and be less influential on research conduct, for those who do not adhere to the normative ideals of science. On this basis, we generate two interaction terms, interacting commitment to scientific ideals of science on a) lack of awareness as to whether their institution has written statement on research integrity, and b) an awareness that their institution does not have a written statement on research integrity.

### Analysis strategy

To account for the non-independence of responses to the QRP items within organisations and countries, and in recognising the hierarchical nature of the data (researchers nested within organisations and countries), we use a multilevel regression approach, implemented using the lme4 package in R (version 2022.12.0 + 353). Adopting a multilevel modelling approach means that we can partition the variability of QRP engagement across individuals, organisations, and countries, allowing us to examine the relative importance of these three components.

Our modelling strategy is as follows. In Model 1 we specify a random intercept model without any predictors, allowing us to assess the variability in QRP reporting at each of our three levels. In Model 2 we again specify a random intercept model, regressing standardised mean QRP engagement on individual-level predictors (incl. contract type, career stage, disciplinary field, and sex). In Model 3 we include our individual-level variable of primary interest, commitment to the normative ideals of science. In Model 4 we include organisation-level predictors (incl. workplace type, integrity breaches, integrity training and RI statement awareness), allowing us to see whether variability in QRP engagement between organisations is explained by these specific organisation-level attributes.

In Model 5 we add our final variable of interest, working environment, into a random-slope model. Including random slopes permits us to see how the relationship between QRPs and a) normative ideals of science, and b) working environment, varies across organisations. Finally, in Model 6 we include several single-level and cross-level interaction terms, allowing us to see whether the relationship between a) commitment to the normative ideals of science, and b) working environment, is moderated by career stage, contract type, RI statement awareness, integrity training and integrity breaches. All models are multiple-linear regression models using restricted maximum likelihood (REML). We have made the full R code available on the Open Science Framework (OSF) [46].

## Results

### Commitment to the normative ideals of science

**Fig. 3:**
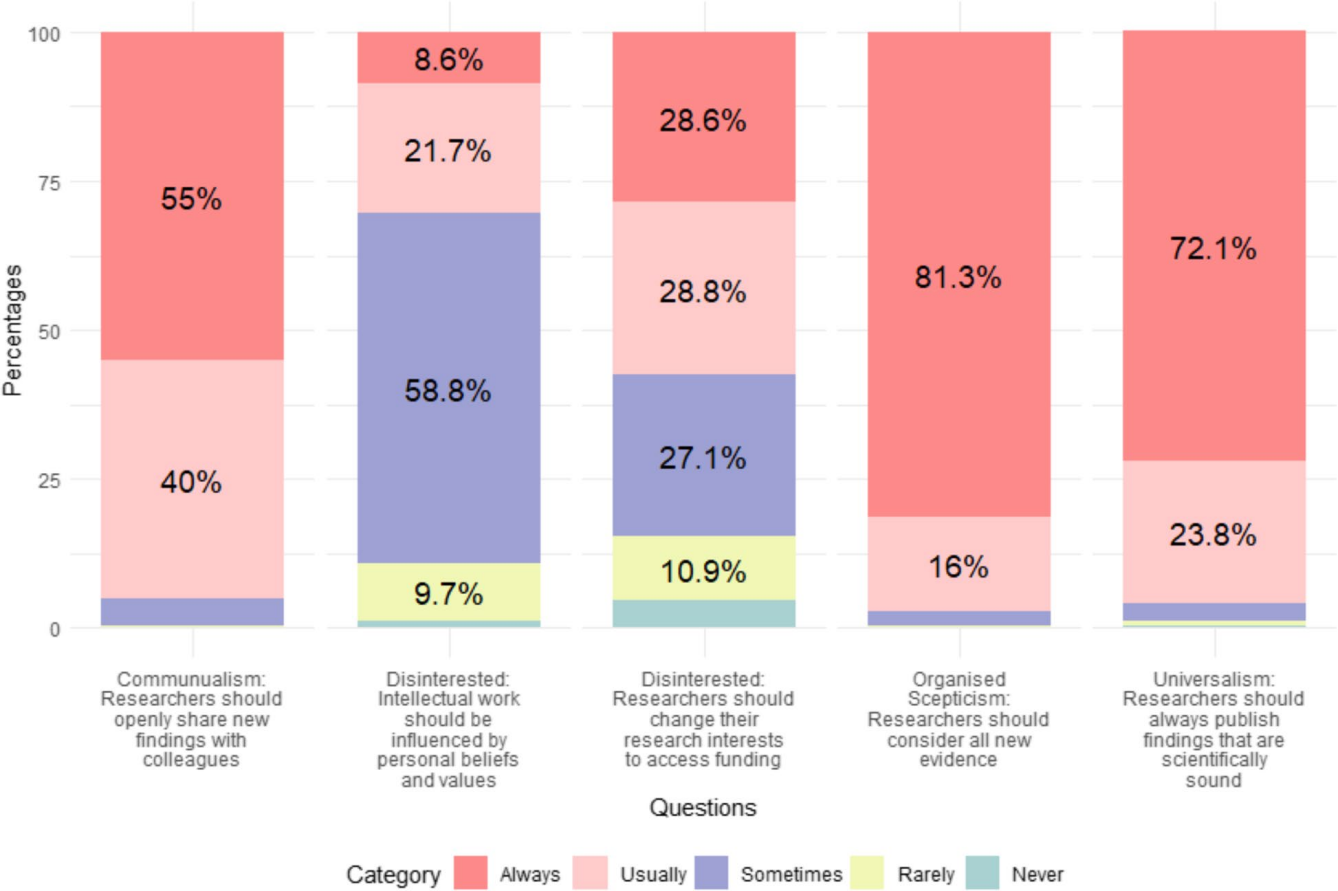
Commitment to normative ideals of science: weighted percentages

Figure 3 shows the weighted percentage of respondents who support a specific practice that undermines or exemplifies the normative ideal of science, indicative of their level of commitment to these norms. A small majority of researchers are demonstrably committed to the normative ideals of communalism, organised scepticism, and universalism, with most respondents agreeing that researchers should always or usually engage in behaviours that are indicative of support to these ideals. Support for disinterestedness is less clear, with a small majority stating that researchers should change their research interests to access funding, and a sizeable majority stating that at least sometimes intellectual work should be influenced by personal beliefs and values. In line with previous research in this area, support is not universal, with a small but significant minority failing to demonstrate commitment to the normative ideals of science.

### Multi-level modelling

Table 3 shows the model fixed effect estimates for mean frequency of engagement in questionable research practices (QRPs) for Models 2 to 6. Model's 2 and 3 show the individual-level fixed effects and random components, while models 4 and 5 introduce our organisational-level predictors. Model 6 includes our interaction terms to test for moderating effects. Model 1 is the intercept only model. The main interest here is in the variance components. The percentage of variance due to country and organisation is extremely small – 0.95% of the variability in average engagement in QRPs is situated at the organisational level and 1.69% of the variability at the country-level. The overwhelming majority of variation in QRP engagement is between individual researchers regardless of where they are.

**Table 3:** Regression coefficients (95% confidence interval) of overall mean questionable research practices (QRP)

	Model 2	Model 3	Model 4	Model 5	Model 6
**Level 1 Fixed Effects**					
Contract Type (ref = permanent)					
no contract	0.09 (0.04, 0.14)***	0.08 (0.03, 0.13)***	0.07 (0.02, 0.12)**	0.05 (0.00, 0.10)*	0.05 (0.00, 0.09)*
temporary contract	0.06 (0.04, 0.09)***	0.06 (0.03, 0.08)**	0.07 (0.05, 0.10)***	0.06 (0.04, 0.09)***	0.06 (0.04, 0.09)***
Career Stage (ref = early career)					
mid-career	-0.05 (-0.08, -0.03)***	-0.06 (-0.08, -0.03)***	-0.07 (-0.09, -0.04)***	-0.07 (-0.09, -0.04)***	-0.07 (-0.10, -0.05)***
later-career	-0.12 (-0.15, -0.09)***	-0.12 (-0.15, -0.09)***	-0.10 (-0.13, -0.07)***	-0.09 (-0.13, -0.07)***	-0.11 (-0.13, -0.08)***
retired	-0.19 (-0.24, -0.14)***	-0.19 (-0.24, -0.14)***	-0.18 (-0.23, -0.14)***	-0.17 (-0.22, -0.13)***	-0.18 (-0.23, -0.13)***
Disciplinary Field (ref = natural sciences)					
medical	0.03 (-0.00, 0.06)**·**	0.05 (0.02, 0.08)**	0.07 (0.04, 0.10)***	0.07 (0.04, 0.10)***	0.07 (0.04, 0.10)***
social	-0.09 (-0.12, -0.07)***	-0.11 (-0.13, -0.09)***	-0.09 (-0.11, -0.07)***	-0.10 (-0.12, -0.08)***	-0.10 (-0.12, -0.08)***
humanities	-0.29 (-0.32, -0.26)***	-0.31 (-0.33, -0.28)***	-0.29 (-0.32, -0.26)***	-0.29 (-0.32, -0.26)***	-0.30 (-0.33, -0.27)***
Sex (Ref = Male)	-0.02 (-0.04, 0.00)**·**	-0.03 (-0.05, -0.01)**	-0.03 (-0.05, -0.01)**	-0.04 (-0.06, -0.03)***	-0.04 (-0.06, -0.02)***
Scientific norms		-0.15 (-0.16, -0.14)***	-0.15 (-0.16, -0.14)***	-0.15 (-0.17, -0.13)***	-0.15 (-0.17, -0.14)***
**Level 2 Fixed Effects**					
Workplace type (ref = academia)					
industry			0.19 (0.13, 0.26)***	0.21 (0.15, 0.27)***	0.21 (0.14, 0.27)***
non profit			0.12 (0.08, 0.16)***	0.14 (0.10, 0.18)***	0.13 (0.09, 0.17)***
government research			0.06 (0.02, 0.10)**	0.07 (0.04, 0.11)***	0.07 (0.03, 0.11)***
health research			0.06 (0.01, 0.11)*	0.07 (0.02, 0.13)**	0.07 (0.02, 0.12)**
other			0.04 (-0.03, 0.10)	0.04 (-0.03, 0.11)	0.04 (0.03, 0.10)
Integrity Breaches			-0.08 (-0.09, -0.06)***	-0.05 (-0.07, -0.04)***	-0.05 (-0.06, -0.04)***
Integrity Training			-0.02 (-0.03, -0.01)***	-0.01 (-0.02, 0.00)	-0.00 (-0.02, 0.01)
Awareness of RI statement (ref = aware)					
unaware			0.02 (0.00, 0.04)*	0.03 (0.01, 0.05)**	0.03 (0.01, 0.05)**
no statement			0.09 (0.06, 0.12)***	0.10 (0.07, 0.13)***	0.11 (0.08, 0.14)***
Working environment				-0.10 (-0.11, -0.08)***	-0.12 (-0.15, -0.10)***
**Interactions**					
Working environment * scientific norms					0.01 (0.00, 0.02)*
Scientific norms * integrity training					-0.02 (-0.03, -0.01)***
Working environment * integrity training					-0.01 (-0.02. 0.00)**·**
Working environment * integrity breaches					-0.01 (-0.02, 0.01)
Working environment * mid-career					0.05 (0.03, 0.08)***
Working environment * later-career					0.06 (0.03, 0.09)***
Working environment * retired					0.06 (0.02, 0.11)**
Working environment * temporary contract					-0.03 (-0.06, -0.01)**
Working environment * no contract					-0.03 (-0.08, 0.02)
Scientific norms * integrity breaches					-0.00 (-0.01, 0.01)
Scientific norms * no statement on RI policy					-0.04 (-0.07, -0.01)*
Scientific norms * unaware of RI policy					0.03 (0.01, 0.05)**
Working environment * unaware of RI policy					-0.01 (-0.03, 0.02)
Working environment * no statement on RI policy					-0.00 (-0.03, 0.03)

Source: International Research Integrity Survey (2021); *n* = 39,699, organisation = 7,666; country = 34; restricted maximum likelihood estimation; unweighted

p < 0.05, * p < 0.01, ** p < 0.001, *** p ≈ 0

Overall mean QRP was computed as an average score based on the 8 QRP items with not applicable scores removed

Model 2 introduces individual-level predictors. Researchers in the social sciences (β = -0.09) and humanities (β = -0.29) engaged in fewer QRPs on average in comparison to those in the natural sciences. In comparing the medical sciences to the natural sciences, there was a smaller yet statistically significant difference in the engagement with QRPs (β = 0.03). Individuals in the middle of their careers (β = -0.05), those later in their careers (β = -0.12) and those who are retired (β = -0.19) reported engaging in fewer QRPs on average in comparison to those early in their careers. A significant difference in average number of QRPs engaged in was observed between individuals without an employment contract (β = 0.09) and those on a temporary employment contract (β = 0.06), compared to those with a permanent contract. That is, those without an employment contract or those on a temporary contract engage in a greater number of QRPs on average compared to those on a permanent contract. However, the effect size is very small, indicating that the practical impact of type of employment contract on engagement in QRPs is relatively minor. Small sex differences are present, with women being less likely to report QRPs (β = -0.02).

Of primary interest in this paper is the extent to which a researcher's commitment to the normative ideals of science is associated with the researchers engagement in QRPs. In Model 3, our composite indicator representing researchers' commitment these normative ideals was added. As predicted, a greater commitment to the normative ideals of science has a negative association with the standardised average number of QRPs engaged in (β = -0.15), while holding other predictors constant.

Organisational-level predictors were added as covariates in Model 4. Average QRP engagement was observed to be greater among individuals working in industry (β = 0.19) and non-profit organisations (β = 0.12), government research centres (β = 0.06) and health research centres (β = 0.06), in comparison to those working in academia. There was no relationship found between researchers’ perceptions of one's environment more closely resembling one that provides research integrity training and average engagement in QRPs. The presence of procedures and qualified staff to handle research integrity breaches was negatively related to average number of QRPs engaged in (β = -0.08). Having no awareness of whether one's institution has a written statement on research integrity is associated with a higher average engagement in QRPs (β = 0.02). Reporting that an individual's institution does not have a written statement of research integrity is associated with a higher average engagement in QRPs (β = 0.09). Again, the magnitude of the effect sizes is small. The data suggests that a researcher's normative commitment to the ideals of science robustly predicts the extent of reported engagement in QRPs. This is more pronounced than the influence of factors such as provision of research integrity training at one's institution, the existence of procedures and qualified staff to address research integrity breaches, or an awareness of a research integrity statement. Of note here is the resilience of a researcher's commitment to normative ideals against all other covariates that are introduced.

**Fig. 4:**
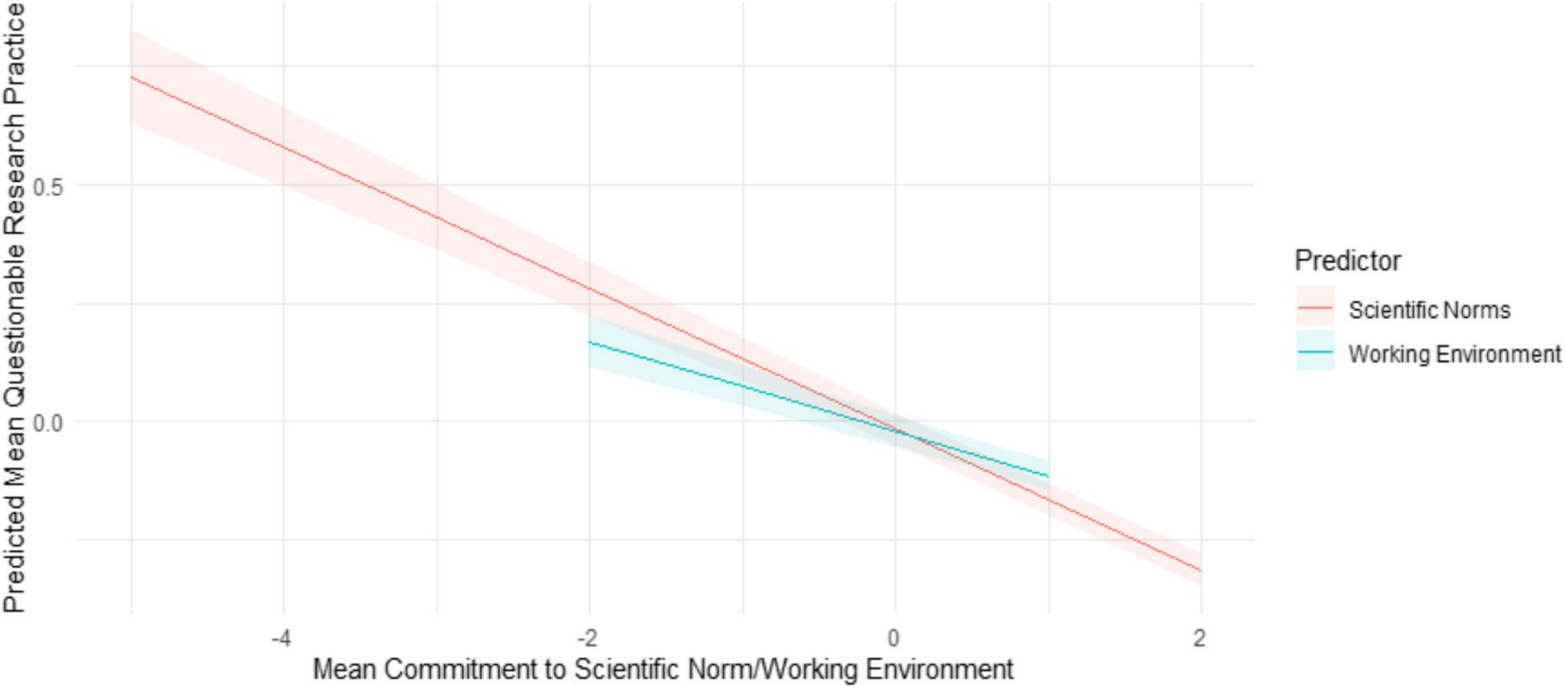
Predicted marginals plot showing effects of working environment and scientific norms on questionable research practices

The extent to which the perceived collegiality and degree of harmful publication pressure affects engagement in QRPs was of particular interest to us. On this basis, the extent to which an environment was perceived to be collegial, without harmful publication pressure, harmful power disparities and conflict was added as a covariate in Model 5. The more collegial an individual's working environment is, the less individuals report engaging in QRPs (β = -0.10). Figure 4 illustrates predicted mean QRP engagement according to both commitment to scientific norms and the extent to which a researcher’s working environment is perceived as collegial, our predictors of particular interest. The x-axis shows a range of standardised values for both of these predictors, with the red and blue lines representing norms and environment respectively. The y-axis represents the standardised mean QRP engagement. A notable negative relationship between QRP engagement and both of these predictors is visible. This suggests that researchers who adhere more closely to scientific norms tend to engage less in QRPs. Moreover, the extent to which a researcher's environment is perceived as collegial and free from harmful publication pressure, shows a negative association with QRP engagement. Researchers working in more supportive and less pressured environments tend to report engaging less often in QRPs.

Model 6 tests for selected moderation effects. A significant interaction between an individual's perception of their working environment and those who are retired (β = 0.06), those late in their career (β = 0.06) and those in the middle of their career (β = 0.05), in comparison to those early in their career, was observed. These estimates indicate that the extent to which an environment is collegial—free from harmful publication pressure, harmful power disparities and conflict—is a more critical factor in mitigating extent of QRP engagement for early-career researchers compared to mid-career, late-career, or retired researchers. Likewise, and unsurprisingly, the harmful nature of the working environment matters more for those on temporary employment contracts (β = -0.03) in comparison to those with permanent contracts. However, and perhaps surprisingly, we found that the extent to which the working environment is perceived as collegial and free from publication pressure has no differential association with QRP engagement for those with no employment contract relative to those on a permanent employment contract.

A significant interaction was observed between the extent to which a researcher is committed to the norms of science and whether they have no awareness of whether their institution has a research integrity statement. We found that for those who are unaware of whether their institution has a written statement on research integrity, a stronger relationship exists between extent of commitment to scientific norms and extent of engagement in QRPs (β = 0.03). That is, a scientist’s commitment to norms of science matters more when a researcher is unaware of whether their institution has a statement on research integrity. Conversely, when a researcher is aware that their institution does not have a research integrity statement, a researcher's commitment to the norms of science has less of an influence on the extent to which they engage in QRPs (β =—0.04).

For researchers with a stronger commitment to the norms of science, being in a working environment that heavily emphasises research integrity training is related to a slight reduction in engagement in QRPs (β = -0.02). Research integrity training contributes to reduced engagement in QRPs in environments not otherwise characterised by publication pressures, unequal power imbalances or non-collegial relationships (β = -0.01). Surprisingly, for those working in a more collegial environment, that is free from harmful publication pressure and power imbalances, a stronger commitment to the norms of science is associated with a slight increase in engagement in QRPs (β = 0.01).

Turning to the random components, the variance explained by each of the grouping variables, organisation, and country, is very weak and relatively consistent across models. Table 4 shows the random effect estimates for mean frequency of engagement in QRPs for models 2 to 6. Models 1–4 are random intercept models, while models 5 and 6 are random slope models. We found that the percentage of variance explained by organisation is 0.87% and by country is 1.05%, indicating that 0.87% and 1.05% of the residual variability in QRP engagement can be attributed to differences between organisations and countries, respectively. The estimated intercept variance for organisation was 0.01 across all models, indicating that only a small amount of variability in extent of QRP engagement can be attributed to organisational differences, after accounting for other predictors in the model. Likewise, the estimated intercept variance for country was also found to be 0.01 in most models, suggesting that only a tiny amount of the variability in QRP engagement can be attributed to differences between countries. This explained variance is robust to the inclusion of group-level predictors into the model, with limited change between the null model and Model 4.

**Table 4:** Random effects of overall mean questionable research practices (QRP)

	Model 2	Model 3	Model 4	Model 5	Model 6
Variance (Working Environment | Organisation)				0.01	0.01
Variance (Scientific Norms | Organisation)				0.02	0.02
Variance (Working Environment | Country)				0.00	
Variance (Scientific Norms | Country)				0.00	
Variance country (Intercept)	0.02	0.01	0.01	0.01	0.01
Variance Organisation (Intercept)	0.01	0.01	0.01	0.01	0.01
Variance (Residual)	0.84	0.82	0.81	0.78	0.80
% of country-level variance	1.69%	1.53%	1.05%		
% of organisation-level variance	0.95%	0.78%	0.87%		

Source: International Research Integrity Survey (2021); *n* = 39,699, organisation = 7,666; country = 34; restricted maximum likelihood estimation; unweighted

Moreover, there was minimal variability in the extent to which commitment to scientific norms and perceptions of one's working environment influenced QRP engagement. That is, the relationship remained consistent across organisations and countries. This variance is additionally robust to the inclusion of single-level and cross-level interactions in Model 6.

## Discussion

The IRIS is the largest survey of its kind. Respondents represent researchers from across all disciplinary fields and a large number of research institutions across Europe and USA. As such, the survey data allow for a more comprehensive analysis of support for the normative ideals of science and an exploration of the relationship between widely postulated individual- and organisational-level factors and QRP engagement than has thus far been possible. We find that self-reported commitment to each of the normative ideals of science is widespread, but not universal. A small majority of respondents state that researchers should sometimes change their research interests to access funding, while a substantial minority believe that intellectual work should be influenced by personal beliefs and values. This finding of non-universal, but widespread, adherence is in line with findings from other survey research [25–28]. While minor divergences from these norms may at first appear benign, they can, as the present research suggests, have consequences for behaviour.

One of our most surprising findings is that only a very limited amount of variance in QRP engagement can be attributed to differences between organisations and countries. This, to us, suggests that factors beyond the organisational and national contexts play a significant role in influencing QRP engagement and, at the same time, that idiosyncratic researcher differences are much more important than the local contexts in which they work. What follows from this is that perhaps more emphasis should be placed on the broadest systemic-level factors that transcend research institutions, such as hyper-competition and publication pressures, compounded by an editorial bias for the aesthetic quality of results, as well as individual-level factors. In considering the individual-level factors captured in our analysis, we find some disciplinary-level differences, with those researching in the arts and humanities engaging in fewer QRPs on average, compared to all other disciplines. This aligns with the findings of Schneider et al. [19] However, contrary to Schneider et al., [19] and additionally Gopalakrishna et al., [29] Fanelli [9], and Pupovac and Fanelli [47], we find that those in the medical sciences and the natural sciences engage in more QRPs on average compared those in the social sciences. Moreover, our findings conflict with the findings of Xie and colleagues [10] who find that QRPs are more common in the social sciences compared to the biomedical sciences. To the extent that researchers’ commitments to responsible research practice is in part due to their training and positioning within their disciplines, it is here that the potential for change lies.

Those who are later in their career admit fewer QRPs on average, again consistent with Schneider et al. [19]. We find this on the one hand unsurprising, given that those later in their career are likely under reduced pressure to publish and obtain funding (having already secured permanency and established careers) and considering evidence to suggest that career-motivations may become less salient with age [48]. That is, the influence of systemic pressures may become less salient with academic age. On the other hand, those that did their graduate training a long time ago would have had less (or no) emphasis placed on what are now known to be detrimental practices – for instance p-hacking. So it is somewhat surprising that the same senior researchers report fewer QRPs.

Affording additional support to the reduced pressure with seniority theory, we find that those on temporary employment contracts or without an employment contract are more likely to engage in QRPs on average, suggesting that precarity can motivate researchers to engage in questionable practices, feasibly to confer an advantage on them (i.e., increased likelihood of publication) where competition (i.e., for employment) is heightened. That is, they have 'more to gain', compared to their permanently employed colleagues.

Relatedly, the organisational-level factor, indicating the extent to which the researcher’s working environment is perceived to be collegial, without harmful publication pressure, detrimental power imbalances and conflict, is associated with decreased average QRP engagement. This finding is not surprising, and again in-part supports dominant theorising that hyper-competitive working environments with harmful publication pressure motivate engagement in QRPs [30]. It aligns with similar research findings focusing on samples from specific disciplinary fields and countries [29, 37, 49, 50], as well as Schneider et al.'s [19] recent cross-national survey of researchers from across disciplinary fields and national contexts. However, the item presented in IRIS captures a more holistic view of the research environment (perhaps, indicative of local research culture), potentially diluting the specific effect of publication pressure itself. It suggests a complex interplay of factors that contribute to unethical behaviour, inclusive of, but not limited to, publication pressure. This conceptualisation additionally aligns with earlier survey research by Schneider et al., [19] who found that local culture that promotes quality and rigorous research, and rewards integrity, potentially counter presumed systemic challenges. On this basis, our finding underscores the importance of fostering a collaborative and supportive research culture, where emphasis is placed on collaboration, peer support and scientific integrity, rather than aggressive competition and high publication output. In cultivating such a research culture, it may be possible to mitigate engagement in QRPs and enhance the quality of scientific research. Given that we found limited differences in QRP engagement between institutions, this explanatory factor likely supports the narrative of corrupting systemic structures that transcend organisational working environments. That is, the issue is likely system-wide, and not unique to particular institutions.

We find sex differences consistent with earlier research. Women appear to engage in somewhat fewer QRPs on average in comparison to men. Similarly, Gopalakrishna et al. [29] found prevalence to be higher among males, in comparison to females. Schneider et al. [19] found a similar relationship in their Danish sample, with men engaging in slightly higher rates of QRPs compared to women, but this finding was less certain in their international sample. More generally, our findings here align with research showing that males have lower moral standards in competitive environments [51], are more likely to deceive where the deception benefits the deceiver [52, 53] and are less risk averse [54].

Engagement in QRPs was higher, on average, in research institutions outside academia, including health and government research centres, private industry and non-profit organisations. Those working in 'industry' were more likely to engage in QRPs on average compared to all other sectors. This aligns with research findings that suggest researchers with private industry involvement are more likely to report engaging in research misbehaviours [55]. While the exact reasons for our findings on this are a matter of speculation, it is possible that factors other than the pursuit of knowledge and scientific inquiry (e.g., a researchers' values and goals) may influence a researcher’s decision-choices. These priorities may overshadow the commitment to strict scientific method and considerations of validity and reliability, inadvertently encouraging QRPs. For example, in government and health research centres, there may be significant pressure to produce results that align with policy objectives and public health goals. Researchers working within these settings may be motivated to take shortcuts to demonstrate immediate and impactful outcomes. Again, considerations that are antithetical to the scientific enterprise may motivate a researcher to cut corners.

Subscription to the normative ideals of science was the explanatory factor scale with one of the largest correlates of decreased QRP engagement. This suggests that a researcher's ideals and normative values may act as a safeguard against other motivating factors and be powerful drivers of researcher decision-making. This corroborates findings by Gopalakrishna et al. [29], who found that commitment to scientific norms was the largest indicator of QRP engagement. Building on recommendations made by Gopalakrishna and colleagues [29], as well as earlier findings from Anderson and colleagues [25], our findings here suggest that it is crucial for institutional leaders to foster a culture that upholds and respects the normative ideals of science. Through formalised training, adequate mentoring and supervision, commitment to these ideals can be encouraged.

Conversely, our findings show that the mere provision of research integrity training does not predict decreased engagement in QRPs. However, it is crucial to note that this measure reflects the 'availability of research integrity training' at one's institution, rather than active engagement by researchers. Certainly, research showing that integrity training can influence integrity is contested and weak [56], see [57–59] while early-childhood education and personality traits have been found to be better predictors of researcher behaviour than research integrity training [56]. A more reasonable conceptualisation is that the availability of training may signify an organisational culture that values research integrity. However, as we find here, the presence of integrity training alone is not necessarily enough to deter researchers from engaging in QRPs. In contrast to this finding, we find a negative association between QRP engagement and the presence of an environment that is perceived to be supportive, characterised by expertise for dealing with integrity breaches, effective mechanisms for detecting and sanctioning breaches, and protections for whistle-blowers and those accused of circumventing integrity standards. While it is not possible to disaggregate the relative influence of each component, it suggests that fostering a research environment with comprehensive support for research integrity (including expertise, monitoring, and protection), beyond the mere provision of research integrity training, may be efficacious in reducing QRP engagement to some degree. It is worth nothing that this represents a researcher’s perceptions of their working environment, rather than representing a true description of the institutional working environment. Additionally, individuals within the same institution might perceive conditions, policies, and structures differently. They may also have varying levels of awareness regarding the existence of these conditions, policies, and structures.

### Limitations

Overall, our effect sizes are, while coherent and consistent with much of the extant literature, rather small. We should be concomitantly humble in our interpretation of the results.

A limitation of our study, not necessarily unrelated to the foregoing is that the QRPs used in the survey were selected non-randomly, and represent a distinct set of practices that qualitatively differ from some of the practices explored in other surveys. They were designed to capture behaviours that could apply equally to all kinds of research – quantitative, qualitative, and theoretical. This means that they might be regarded as a somewhat blunt tool, compared with discipline specific studies such as John et al. [1] that have only one disciplinary focus.

One further limitation is that the sample focused primarily on countries in Europe and North America. On this basis, it is not possible to generalise our findings beyond Europe and the Anglo-American sphere.

## Conclusion and future research

Our research contributes to the explanatory framework of QRP engagement, identifying both systemic and individual-level factors that predict, albeit weakly, self-reported QRP engagement. Given the lack of institutional variance, we suspect that the system-wide pressures (to publish frequently, in high-impact journals and to obtain funding) is a major contributing factor to QRP engagement and acts directly on the individual researcher. Institutional mediation of such pressure, if it occurs, does not appear to be a strong channel. This is an interesting thought, if a little concerning, because it is easier for organisations to change than it is for systemic change to be brought about ‘top down’.

Commitment to the Mertonian norms of science seem to protect to some extent against the systemic and other individual-level influences. Studies that examine the impact of other individual-level characteristics in reducing engagement in QRPs highlight factors such as personality traits [19], achievement goals [22], perceived ethical defensibility, and perceptions of what is considered normative behaviour in the field [23]. To further understand this and build a more comprehensive explanatory model, future research should focus on identifying other individual-level factors that might impact researcher behaviour. For example, a researcher's personal reasons behind their research decisions, such as social or political goals, financial improvement, concerns about validity and reliability, along with motivations directly linked to the scientific process like obtaining grants, increasing frequency of publication, desiring novelty, societal impact, and the replication of results, could affect their behaviour and their willingness to compromise on research integrity. A more explicit analysis of how these individual-level factors protect against, or enhance, systemic-level causes is required, and will allow for a fuller, more comprehensive explanatory model for researcher QRP engagement. This will permit the development of evidence-based interventions and policies that are efficacious in reducing QRP engagement, improving research integrity, and creating a more valid, reliable, and trustworthy science.

## Supplementary Information

Supplementary Material 1. Figure S1. Weighted frequency of respondents by organisation (*n* = 7,666).

## Data Availability

The code used for the analysis in this study is openly available on the Open Science Framework: https://osf.io/sg8zf/. The primary dataset utilised for this study can be found at the UK Data Service: https://beta.ukdataservice.ac.uk/datacatalogue/studies/study?id=9023. The organisational dataset analysed during the current study are not publicly available due to identifying information but are available from Nick Allum upon reasonable request.

## Abbreviations

QRPs: Questionable Research Practices
LMIC: Low-income and middle-income countries
HIC: High-income countries
