# Supplementary material for: Investigating the links between questionable research practices, scientific norms and organisational culture
Source: Res Integr Peer Rev. 2024 Oct 14;9:12. doi: 10.1186/s41073-024-00151-x (PMC11472529; doi:10.1186/s41073-024-00151-x)
Supplement: Supplementary file 1 — Supplementary Material 1. Figure S1. Weighted frequency of respondents by organisation (n = 7,666). [file 41073_2024_151_MOESM1_ESM.pdf]

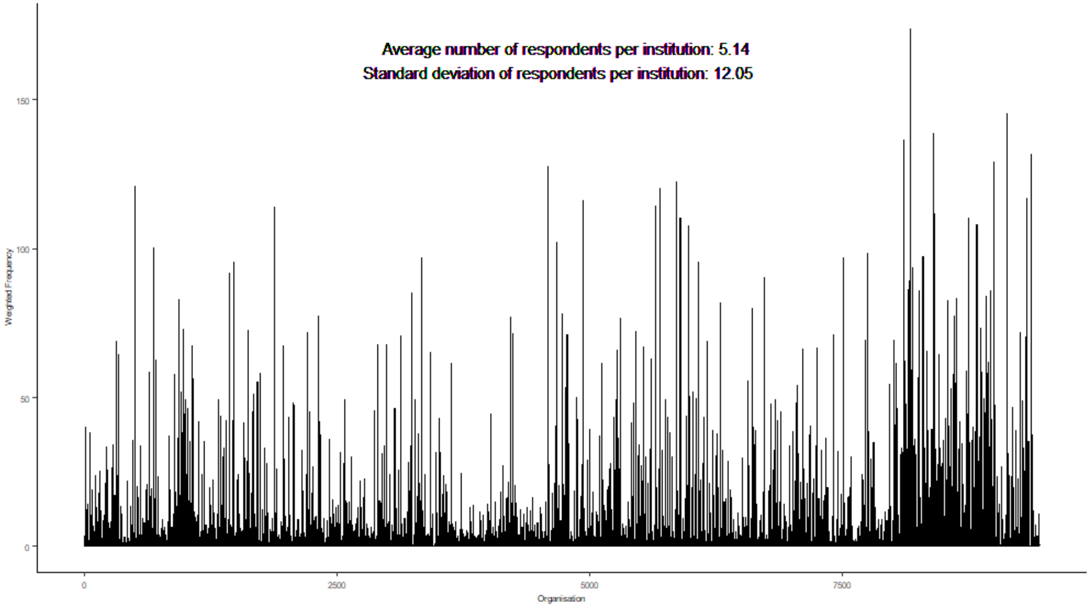
Average number of respondents per institution: 5.14
Standard deviation of respondents per institution: 12.05
150
100
50
0
Weighted Frequency
0
2500
5000
7500
Organisation
